# Combination of Navigated Macular Laser Photocoagulation and Anti-VEGF Therapy: Precise Treatment for Macular Edema under Dry Retinal Conditions

**DOI:** 10.1155/2017/7656418

**Published:** 2017-02-20

**Authors:** Ernest V. Boiko, Dmitrii S. Maltsev

**Affiliations:** ^1^St. Petersburg Branch of the Academician S. Fyodorov IRTC “Eye Microsurgery”, 21 Yaroslav Gashek St., Saint Petersburg 192283, Russia; ^2^Department of Ophthalmology, Mechnikov North-West State Medical University, 47 Kirochnaya St., Saint Petersburg 191015, Russia; ^3^Department of Ophthalmology, Military Medical Academy, 5 Klinicheskaya St., Saint Petersburg 194044, Russia

## Abstract

*Purpose*. To compare the controllability of navigated macular laser photocoagulation (MLP) in dry versus edematous retina and validate that pretreatment diagnostic images can be used as basis for navigated MLP after the macular edema (ME) has been resolved. *Materials and Methods*. Group 1 was divided into subgroup 1 (dry retina MLP) and subgroup 2 (MLP in ME) for comparisons of laser-burn diameters. In group 2, the areas and locations of ME before an intravitreal injection of anti-VEGF (IVAV) were compared with those of recurrent ME. *Results*. The average actual diameter as percentage of planned diameter of laser burn in subgroup 1 (11 DME eyes, 6 BRVO eyes) versus subgroup 2 (5 DME eyes, 8 BRVO eyes) was 115.1 ± 9.1% versus 167.2 ± 13.8% (based on retro-mode scanning laser ophthalmoscopy), and 118.1 ± 14.8% versus 176.1 ± 11.6% (based on OCT) (*p* < 0.001). In group 2 (6 DME eyes, 6 BRVO eyes), difference in mean ME area before IVAV and that in recurrent edema was insignificant (*p* > 0.05). *Conclusion*. The controllability of navigated MLP in dry retina is improved compared to edematous retina. This study validates that pretreatment diagnostic images can be used as basis for navigated MLP after the edema has been resolved.

## 1. Introduction

In diabetic macular edema, the combination of navigated macular laser photocoagulation (MLP) and antivascular endothelial growth factor (VEGF) therapy seems to reduce the number of injections needed with comparable good visual outcome as anti-VEGF monotherapy [1, 2]. In addition, MLP is indicated also for branch retinal vein occlusion (BRVO) without spontaneous resolution of macular edema [3–5].

MLP is performed in the regions of vascular leakage which are associated with retinal edema and thickening and are demonstrated by fluorescein angiography (FA) [6]. In recent years, optical coherence tomography (OCT) [7] and retro-mode scanning laser ophthalmoscopy (RM-SLO) [8] have been used to identify the retinal regions requiring photocoagulation. Unfortunately, in the presence of significant retinal edema and thickening, conventional MLP has substantial limitations due to difficulties in delivering precise amounts of laser energy to treatment areas.

Laser beam diffusion and difficulties with efficient visualization of laser burns due to thickened neurosensory retina may result in development of oversized laser burns and, consequently, excessive chorioretinal scars and imminent atrophic creep of the retinal pigment epithelium [9]. MLP would be performed more easily, with improved control by the physician, if it could be preceded by either partial or complete resolution of macular edema (ME) with intravitreal anti-VEGF therapy or steroids. The use of MLP under dry retinal conditions requires, however, preliminary planning, because, after ME resolution, FA shows reduced or no regions of vascular leakage [10], whereas OCT shows reduced or normal retinal thickness [11].

Precise planning for MLP with the use of different methods for determining the boundary of retinal edema has become possible with the help of navigated technology. The latter involves superimposition of FA image, OCT thickness map, or RM-SLO image onto a color fundus photograph and placing laser spot marks at the regions of retinal vascular leakage, retinal thickening, or retinal edema [8, 12]. It is, however, unclear whether the area and location of recurrent retinal edema differ from those of pre-anti-VEGF treatment edema. Answering this question may help perform MLP in the most appropriate way under dry retinal conditions.

The purpose of this work was to compare the controllability of navigated MLP in dry versus edematous retina and validate that pretreatment diagnostic images can be used as base for navigated MLP after the edema has been resolved. The key indicator of MLP controllability was the comparison of (a) planned and actual laser burn diameters and (b) the laser power required for induction of laser burns in navigated MLP in the presence of macular edema with that after resolution of edema following treatment with a single intravitreal injection of anti-VEGF agents (IVAV). To validate the use of a pretreatment diagnostic image as base for navigated MLP, the area and location of ME before anti-VEGF were compared to those of recurrent ME after anti-VEGF when no laser had been used.

## 2. Materials and Methods

The study was approved by the Ethics Committee of Military Medical Academy and followed the tenets of the Declaration of Helsinki. Before treatment, patients were explained the cause of the disease and management options available to address macular edema, as well as advantages and disadvantages of these options. A management plan was agreed with each patient, and subsequent written informed consent was obtained for both participation in the study and for IVAV injection or MLP. Patients' decision in favor of having MLP (instead of anti-VEGF) as the first management stage was free, conscious, and voluntary.

This prospective study included patients with macular edema associated with diabetes (DME) or BRVO-related ME and not treated previously with MLP or anti-VEGF therapy.

**Figure 1:**
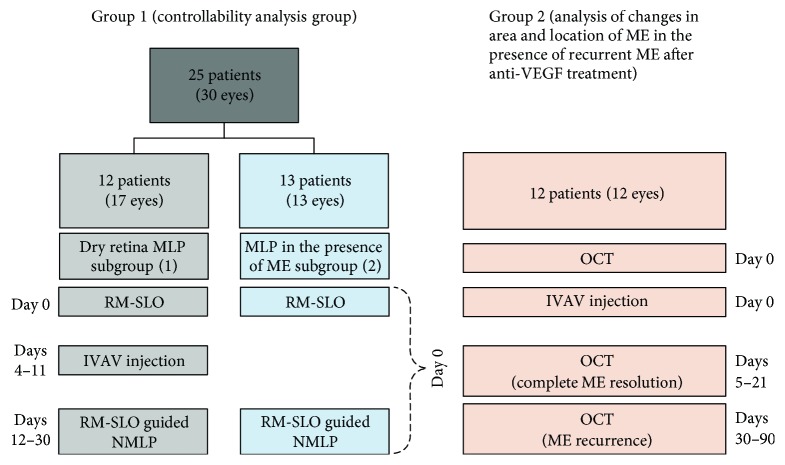
Schematic overview of the groups of the study.

There were two groups of patients. Group 1 (controllability analysis group) was used for comparisons (a) between the planned and actual laser burn diameters and (b) between the average laser power required for induction of laser burns in MLP in the presence of macular edema (subgroup 2) and that after resolution of edema following treatment with a single IVAV injection (subgroup 1). Group 2 was used for comparisons of the areas and locations of ME before anti-VEGF with those of recurrent ME after anti-VEGF (without MLP) (Figure 1).

Exclusion criteria included evidence of acute or chronic uveitis, vitreoretinal traction, fibrosis of the internal limiting membrane (with macular involvement), central RVO, or apparent optic media opacity (including cataract grades 2–4 on the Lens Opacity Classification System scale III [13]). An additional exclusion criterion for patients with branch RVO (BRVO) was duration of BRVO < 3 months. Ranibizumab injections (Lucentis) were administered to patients with DME (0.3 mg/0.05 cc) and to those with BRVO (0.5 mg/0.05 cc) of subgroup 1 and group 2, as per manufacturer's instructions.

### 2.1. MLP Controllability Analysis

At baseline, patients of group 1 (controllability analysis group) underwent RM-SLO and OCT.

The RM-SLO images obtained with SLO F-10 (NIDEK, Gamagori, Japan) were utilized for photocoagulation treatment planning and for measurements of actual diameters of laser burns after photocoagulation.

OCT retinal thickness maps (Enhanced Macular Map 5 (EMM5) protocol) were acquired on the spectral domain OCT system (RTVue-100, Optovue, Fremont, CA) and were used for determination of retinal thickness before MLP treatment planning. 3D reference scan pattern and line scan pattern were used for measurements of actual diameters of laser burns after MLP.

**Figure 2:**
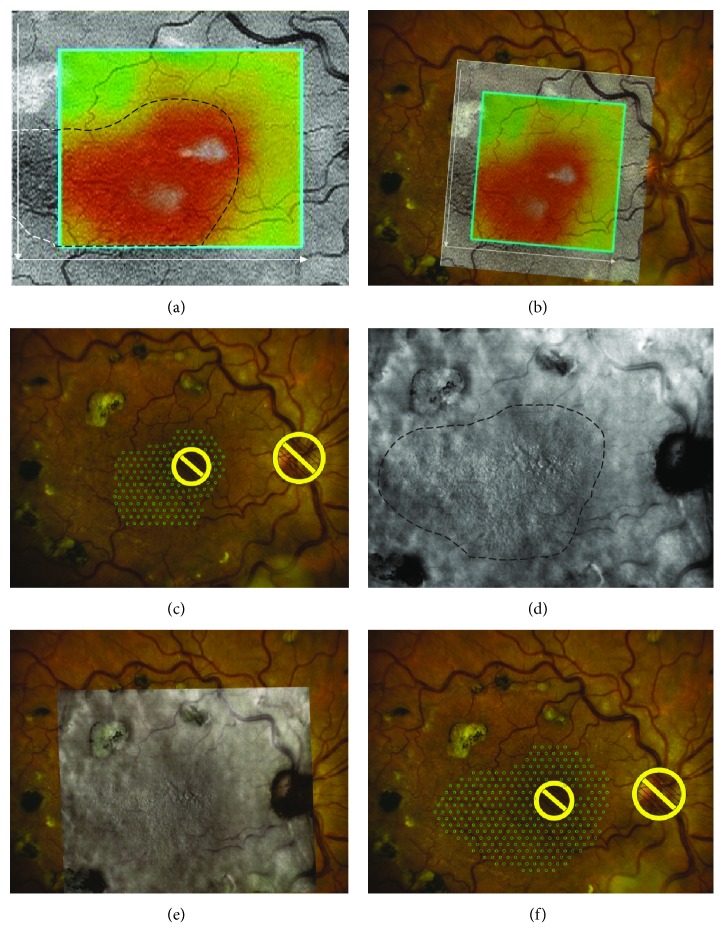
MLP planning based on OCT and RM-SLO. (a) OCT retinal thickness map demonstrates a thickened region (black dashed line) spreading outside the map boundary (white dashed line). (b) OCT map is superimposed onto the baseline image. (c) In OCT-guided planning for macular laser photocoagulation, the number of laser spot marks was 156. (d) RM-SLO image demonstrates a region of retinal edema with numerous microcysts. (e) RM-SLO image is superimposed onto the baseline image. (f) In RM-SLO-guided planning for macular laser photocoagulation, the number of laser spot marks was higher than in OCT-guided planning (259 versus 156).

Navigated MLP was planned and performed using NAVILAS system (OD-OS GmbH, Berlin, Germany). The planning parameters used included a spot size of 100 *μ*m, burn spacing of 2 burn-widths apart, and pulse duration of 100 ms (Figure 2).

Controllability was defined as the conformance between planned and actual diameters of laser burns, with the use of laser power required for the creation of barely visible (light gray) laser burns (as per Early Treatment Diabetic Retinopathy Study (ETDRS)) [6].

In subgroup 1 (dry retina MLP subgroup), MLP was guided by the pre-anti-VEGF RM-SLO and performed after complete resolution of ME following anti-VEGF treatment. Additional inclusion criteria for subgroup 2 were normal central subfield (CSF) retinal thickness values and no intraretinal cysts outside the CSF based on OCT data obtained after a single IVAV injection. After anti-VEGF treatment, eyes were examined on a weekly basis until the resolution of ME. If the requirements above were not achieved during 3 weeks after anti-VEGF treatment, the patient was excluded from the study; this resulted in the dropout of 62.3% of DME patients and 35.5% of BRVO patients. The intravitreal bevacizumab therapy was continued in patients who dropped out due to incomplete resolution of ME following a single injection.

In subgroup 2, MLP was planned and performed in the presence of edema without any preliminary therapeutic treatment.

### 2.2. Comparisons of the Areas and Locations of ME before Anti-VEGF Treatment with Those of Recurrent ME after Anti-VEGF Treatment

Additional inclusion criteria for group 2 were (1) no prestudy history of anti-VEGF treatment, (2) normal CSF retinal thickness values based on OCT data obtained after a single IVAV injection, and (3) recurrent macular edema 1–3 months after a single IVAV injection.

OCT retinal thickness maps (EMM5 protocol) were used for comparisons of the areas and locations of ME before anti-VEGF treatment with those of recurrent (after anti-VEGF treatment) ME. RTVue-100 OCT software was used to quantitate the retinal area exceeding the threshold (350 *μ*m) at full retina thickness maps and determine the area of macular edema. Full Thickness MM5 Significance Maps (related to the significance of the full retinal thickness deviation from normal) were used to compare the location of baseline edema with that of recurrent edema.

**Figure 3:**
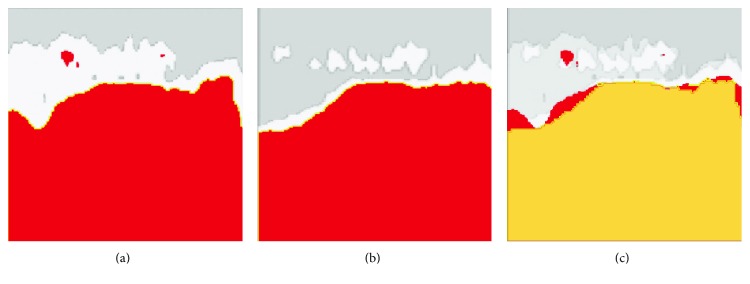
Typical example of the assessment of overlap between locations of initial and recurrent macular edema based on significance maps (related to retinal thickness deviation) in the patient with central retinal vein occlusion. (a) Location of macular edema before anti-VEGF treatment is marked with red. (b) Location of recurrent edema at day 41 after anti-VEGF treatment is marked with red. (c) Region of overlap between locations of initial macular edema and recurrent macular edema is marked with yellow. The region of nonoverlap is marked with red.

The ImageJ software (NIH, Bethesda, MD) was used to measure the area where the retinal thickness value was greater than the 99% confidence limit of normal thickness, both before anti-VEGF treatment and in recurrent ME. The area of overlap between these two areas was determined (Figure 3).

To investigate the degree of conformance between the planned and actual laser burn diameters, we determined the percentage of the ratio of average diameter of 10 laser-induced lesions visualized on RM-SLO (or OCT) 30 minutes after laser photocoagulation, to the planned diameter (100 *μ*m). On OCT, the diameter of laser burn was determined at the outer nuclear retinal layer on the B scan crossing the center of this burn.

Average laser power was determined from the report provided by the Navilas laser system after completion of each laser application.

Statistical analysis was performed with Statistica 10.0 (Statsoft, Tulsa, OK). Unless otherwise stated, all the data are expressed as the means and standard deviation (SD). The Mann-Whitney *U* test was used to assess intersubgroup differences in age, actual diameter of laser-induced burns, and power required to induce a laser burn. A *p* level of 0.05 was considered statistically significant.

## 3. Results

### 3.1. MLP Controllability Analysis

Twenty-one Caucasian patients were included into group 1 (controllability analysis group), with 12 patients (17 eyes; 7 women and 5 men; mean age: 59.2 ± 11.5 years) in subgroup 1 (dry retina MLP subgroup) and 13 patients (13 eyes; 8 women and 5 men; mean age: 62.8 ± 12.6 years) in subgroup 2 (MLP in the presence of ME subgroup). The subgroups were not statistically significantly different in age (Table 1).

**Figure 4:**
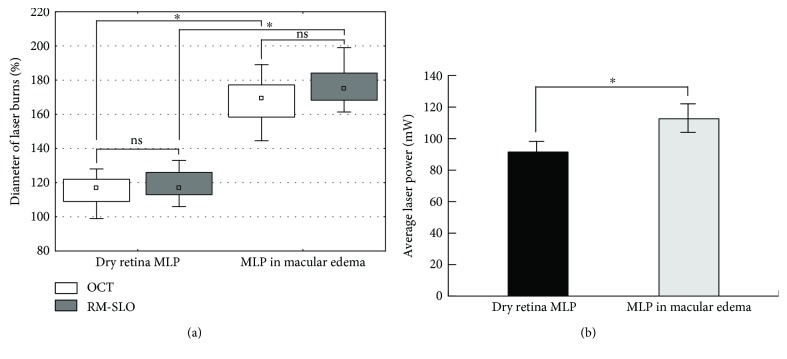
Comparison of the controllability of navigated MLP in dry versus edematous retina. (a) Difference in mean actual diameter of laser burns following navigated MLP in dry versus edematous retina based on RM-SLO (grey boxes) and OCT (white boxes) data. (b) Difference in average laser power for navigated MLP in dry versus edematous retina. ^∗^*p* < 0.05; ns, nonsignificant.

Based on RM-SLO data, average actual diameter as percentage of planned diameter of laser burn in subgroup 1 and subgroup 2 was 115.1 ± 9.1% and 167.2 ± 13.8%, respectively (*p* < 0.001). Based on OCT data, average actual diameter of laser burn in subgroup 1 and subgroup 2 was 118.1 ± 14.8% and 176.1 ± 11.6%, respectively (*p* < 0.001). The intermethod differences in measurements of actual diameter of laser burn were insignificant (Figure 4(a)). There were no statistically significant differences between DME patients and BRVO patients of each subgroup in average actual diameter of laser burns or average laser power (*p* > 0.05) (Table 2).

**Figure 5:**
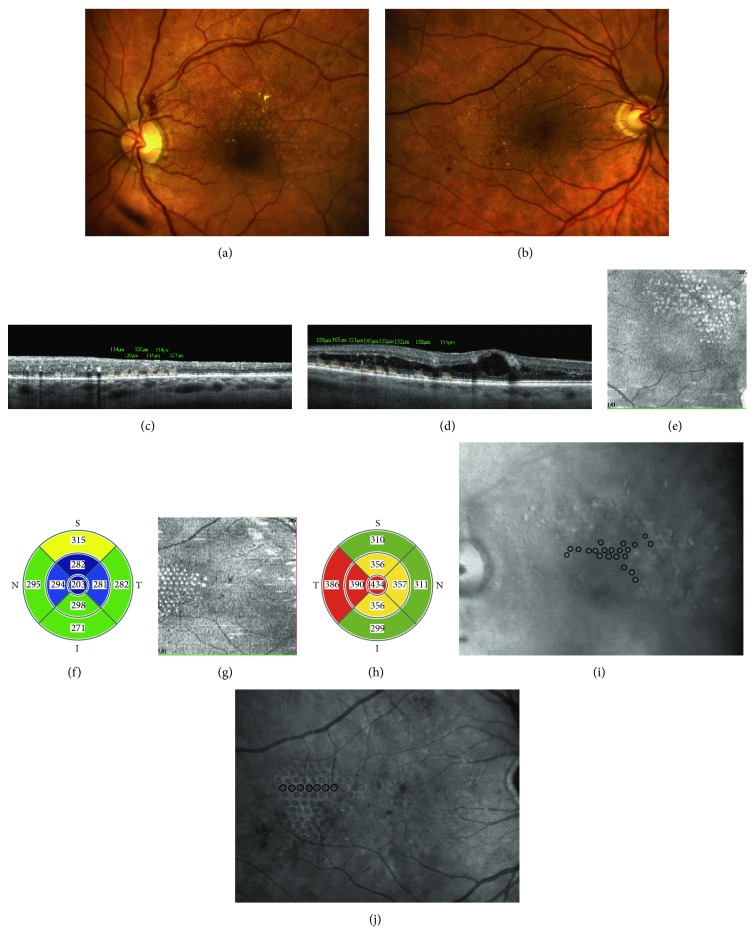
Example of difference in MLP controllability, for dry retinal conditions (subgroup 1) and for the presence of macular edema (subgroup 2). (a) Fundus photograph of the patient (subgroup 1), 30 minutes after MLP. (b) Fundus photograph of the patient (subgroup 2), 30 minutes after MLP. (c) B scan (through a photocoagulated region) of the patient of subgroup 1. (d) B scan (through a photocoagulated region) of the patient of subgroup 2. (e) En face image (at the level of the outer nuclear layer) of the patient of subgroup 1. (f) Retinal macular thickness map demonstrates either normal or decreased retinal thickness values in all ETDRS subfields in the patient of subgroup 1. (g) En face image (at the level of the outer nuclear layer) of the patient of subgroup 2. (h) Retinal macular thickness map demonstrates increased retinal thickness values in all ETDRS subfields in the patient of subgroup 2. (i) RM-SLO image of the patient (subgroup 1) 30 minutes after MLP. (j) RM-SLO image of the patient (subgroup 2) 30 minutes after MLP.

Eyes of subgroup 1 needed less average laser power than eyes of subgroup 2 (91.5 ± 12.3 mW and 112.8 ± 5.4 mW, resp., *p* < 0.01). In subgroup 2 (MLP in the presence of ME subgroup), the increase in actual diameter of laser burn resulted in decrease in burn spacing (Figures 4(b) and 5).

### 3.2. Comparisons of the Areas and Locations of ME before Anti-VEGF Treatment with Those of Recurrent ME after Anti-VEGF Treatment

Twelve patients (12 eyes; 7 women and 5 men; mean age: 64.2 ± 9.5 years) were included into group 2 (analysis of changes in area and location of ME in the presence of recurrent ME after anti-VEGF treatment). Diabetic ME and BRVO-related ME were found in 6 patients (6 eyes) and 6 patients (6 eyes), respectively, of this group.

**Figure 6:**
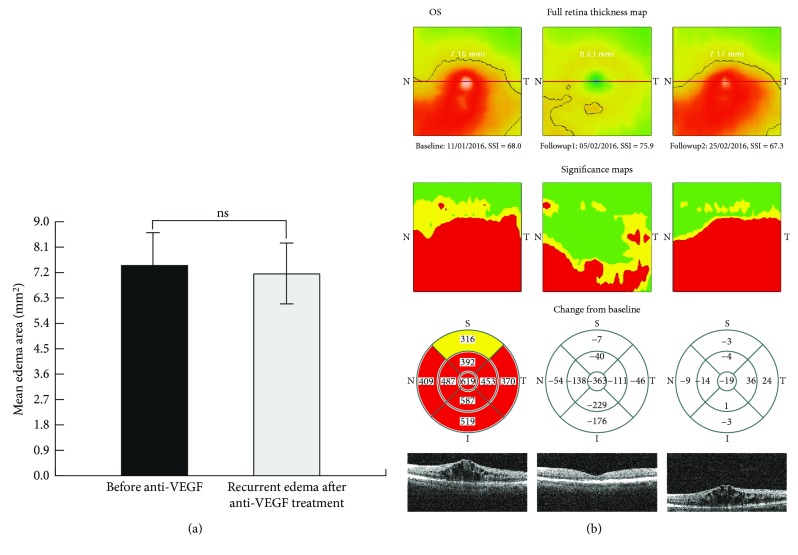
Comparison of the areas and locations of ME before anti-VEGF treatment with those of recurrent ME after anti-VEGF treatment. (a) Difference in mean edema area before anti-VEGF treatment and after ME recurrence. (b) Representative case of recurrent BRVO-related macular edema after a single anti-VEGF injection. Retinal thickness maps demonstrate that the area of recurrent edema was similar to that of edema before anti-VEGF injection. Significance maps demonstrate a near complete overlap between locations of initial edema (before anti-VEGF injection) and recurrent edema. ns, nonsignificant.

The mean edema area before anti-VEGF treatment was 7.45 ± 2.34 mm^2^ and that in recurrent edema after anti-VEGF treatment was 7.15 ± 2.18 mm^2^ (*p* > 0.05) (Figure 6(a)). The mean length of time between anti-VEGF treatment and assessment of edema area in recurrent edema was 45.7 ± 21.9 days. The relative area of overlap between the total edema area before anti-VEGF and that in recurrent edema was 91.6 ± 3.4% (Figure 6(b)).

## 4. Discussion

This study demonstrates that MLP for DME or ME associated with BRVO can be performed with the help of navigated technology in the most precise manner (with the burns more uniform in diameter and in laser power required for their production) under dry retinal conditions, using a retinal macular thickness map obtained before anti-VEGF therapy.

It is known that the thinner the edema, the less laser power it needs in MLP, since accumulation of exudative fluid in macular edema results in reduced retinal clarity and altered penetration of laser radiation into outer retinal layers and into retinal pigment epithelium (RPE) due to power dissipation. Therefore, conventional MLP with gray-white burns around the fovea may cause significant retinal damage in the macula. At the same time, according to ETDRS guidelines, the goal of grid treatment is to create barely visible (light-gray) burns, and absence of visible burns does not allow rating the laser treatment session as being performed adequately [6].

In the present study, we found that an increase by 15–20 mW in laser power was necessary to produce gray-whitish laser burns on retinas that were thickened due to retinal edema. This increase in laser power was found to result in a considerably enlarged size of laser burns at steady laser spot size (100 *μ*m). The uncertainty regarding the resulting size of the laser burn may lead to unfavorable visual acuity or visual field outcome and decreases controllability of the MLP performed in the presence of a retinal edema. Our results go well along with previous studies that demonstrated a higher laser spot application accuracy focal MLP for DME [14] and a higher rate of accuracy in focal MLP treatment of DME than standard manual-technique laser treatment [15]. However, the concordance between the size of actual laser burns and that of planned laser burns has not been investigated until now, and this aspect of MLP controllability and accuracy is more important in grid MLP than in focal MLP due to a higher number of laser burns applied to the retina.

A number of studies have investigated visual outcomes following a combination of intravitreal anti-VEGF [16, 17] or steroid [18] injections with prompt or deferred MLP for macular edema. Although it is clear that intravitreal steroid therapy was performed for reduction of ME and as a pretreatment before MLP [16], no quantitative analysis of MLP controllability was performed, and no retinal assessment was performed on the presence and intensity of retinal edema (expressed as central retinal thickness) at the time of MLP. The use of MLP after resolution of ME will make it possible to avoid the problems of power titration which are associated with edema of the neuroepithelium and to obtain laser-induced retinal burns of required size and spatial distribution.

RM-SLO is the technique which is important to use for accurate measurement of laser burns, since after the MLP performed in the presence of marked macular edema, burn boundaries are poorly defined on the fundus photographs, FA, and even OCT. This is due to the facts that (1) the physical principle used in RM-SLO allows detecting minor elevations of the RPE and medium interfaces (including the border between intact and coagulated retinal tissue) and (2) near-infrared laser radiation emitted by the SLO laser penetrates well through edematous retina. In the study presented here, RM-SLO-based and OCT-based measurements yielded comparable results regarding diameters of laser-induced burns, thus confirming the validity of these findings.

The use of the navigated approach to retinal photocoagulation under dry retinal conditions allows placing laser burns precisely in the locations where the areas of vascular leakage were revealed before anti-VEGF treatment (and where these areas will reappear in the recurrent edema) and avoiding excessive photocoagulation of relatively intact retina.

The use of pre-anti-VEGF edema maps seems reasonable, at least in the short term, since, after a single IVAV injection, the recurrent edema occurs, with its location and area being similar to those of initial edema.

In general, navigated photocoagulation under dry retinal conditions seems to be not only more controllable but also more standardized than conventional MLP.

The results of conventional MLP depend significantly on the intensity of retinal edema (expressed as CRT) and will differ from eye to eye as well as from one retinal subfield to another in the same eye. Theoretically, the problem of excessive actual burn size (compared to the planned one) can be solved for the MLP performed in the presence of edema by reduction of the laser spot size. However, this approach is significantly limited by poor visualization of laser burns and by the difference in edema intensities in different retinal subfields of the same eye. It is the conformance between planned and actual diameters of laser burns which is important for controllability of MLP, whichever diameter is preset. This may be ensured by pre-MLP intravitreal therapy (in particular, in the form of a series of injections) for resolution of edema. In addition, even a partial resolution of ME following IVAV injections may contribute to better MLP controllability, which is important for patients with ME persisting in spite of a series of injections. However, the methodology is not applicable in patients with ME resistant to anti-VEGF therapy. In these cases, anti-VEGF therapy may be either prolonged or stopped and followed by vitrectomy.

The approach described may be also used for subthreshold micropulse laser photocoagulation, especially, for navigated micropulse MLP that has been recently approved for clinical use.

Our study has several limitations. First, the sample size was small (especially in MLP in the presence of ME subgroup) due to strict inclusion criteria followed in the study, as well as due to the fact that most of patients with ME receive intravitreal anti-VEGF therapy as the first therapy in the treatment schedule. Second, the dry retina MLP subgroup included only patients in whom edema resolved completely following a single IVAV injection, whereas patients in whom edema resolved incompletely were excluded. However, it may be also possible to perform MLP in the postedematous period if the number of IVAV injections required for complete resolution of edema is more than one. The requirement for additional data on the area and location of recurrent edema after a series of injections is one of the reasons why we did not investigate this possibility. It is possible that the area and location of recurrent edema after a series of injections will be different from those related to initial edema. Third, in this study, we did not assess functional differences in dry versus edematous retina following navigated MLP. It is well known that laser treatment combined with anti-VEGF results in better functional outcomes in diabetic or BRVO-related ME than does laser monotherapy; therefore, an appropriate comparison for functional outcomes would be to compare ME groups (or subgroups) which received both laser and anti-VEGF. As, in our study, only patients of subgroup 1 received laser treatment combined with anti-VEGF, their functional outcomes should be definitely better than those in patients of subgroup 2. This is the major reason why we have not assessed final visual acuity or other functional outcomes in the study. Fourth, we did not examine late visual and anatomic outcomes. Assessing these outcomes will require a long follow-up, since the improvement after MLP increases slowly, and reduction in central retinal thickness after MLP in some studies has been observed for 2 years [18].

Although the benefits of combination treatment (MLP plus anti-VEGF therapy or intravitreal steroid) for macular edema has been postulated earlier [19], it is possible that “precise laser photocoagulation under dry retinal conditions” proposed will offer additional benefits.

In conclusion, the controllability of navigated MLP in dry retina is improved (with a better concordance between diameters of planned and actual laser burns, and less average power needed) compared to edematous retina. In addition, this study validates that pretreatment diagnostic images can be used as base for navigated MLP after the edema has been resolved.

## Figures and Tables

**Table 1:** Baseline characteristics of the sample.

	Group 1		Group 2
	Subgroup 1	Subgroup 2	
Mean age ± SD, years	59.2 ± 11.5	62.8 ± 12.6	64.2 ± 9.5
Gender (male, *n* (%))	5 (41.7%)	5 (38.5%)	7 (58.3%)
DME patients (eyes), *n*	6 (11)	5 (5)	6 (6)
BRVO patients (eyes), *n*	6 (6)	8 (8)	6 (6)
Mean BCVA ± SD	0.43 ± 0.19	0.21 ± 0.11	0.34 ± 0.15

BCVA: best corrected visual acuity; BRVO: branch retinal vein occlusion; DME: diabetic macular edema; SD: standard deviation.

**Table 2:** Comparison of diameter of laser burn and laser power in DME and BRVO patients.

		Subgroup 1		Subgroup 2	
		DME	BRVO	DME	BRVO
Mean diameter of laser burn ± SD, *μ*m	OCT	112.8 ± 9.2	119.2 ± 8.0	164.8 ± 9.5	168.8 ± 10.2
	RM-SLO	117.9 ± 9.8	118.7 ± 7.1	175.6 ± 11.2	176.4 ± 12.6
Laser power, mW		90.2 ± 13.1	93.0 ± 13.4	111.3 ± 14.8	114.1 ± 16.2

BRVO: branch retinal vein occlusion; DME: diabetic macular edema; OCT: optical coherent tomography; RM-SLO: retro-mode scanning laser ophthalmoscopy; SD: standard deviation.

## References

[1] Barteselli G., Kozak I., El-Emam S., Chhablani J., Cortes M. A., Freeman W. R. (2014). 12-month results of the standardised combination therapy for diabetic macular oedema: intravitreal bevacizumab and navigated retinal photocoagulation. *British Journal of Ophthalmology*.

[2] Liegl R., Langer J., Seidensticker F. (2014). Comparative evaluation of combined navigated laser photocoagulation and intravitreal ranibizumab in the treatment of diabetic macular edema. *PloS One*.

[3] Narayanan R., Panchal B., Stewart M. W. (2016). Grid laser with modified pro re nata injection of bevacizumab and ranibizumab in macular edema due to branch retinal vein occlusion: MARVEL report no 2. *Clinical Ophthalmology (Auckland, NZ)*.

[4] Tadayoni R., Waldstein S. M., Boscia F. (2016). Individualized stabilization criteria-driven ranibizumab versus laser in branch retinal vein occlusion: six-month results of BRIGHTER. *Ophthalmology*.

[5] Tan M. H., McAllister I. L., Gillies M. E. (2014). Randomized controlled trial of intravitreal ranibizumab versus standard grid laser for macular edema following branch retinal vein occlusion. *American Journal of Ophthalmology*.

[6] Early Treatment Diabetic Retinopathy Study Research Group (1987). Treatment techniques and clinical guidelines for photocoagulation of diabetic macular edema. Early treatment diabetic retinopathy study report number 2. *Ophthalmology*.

[7] Schneider E. W., Mruthyunjaya P., Talwar N., Harris Nwanyanwu K., Nan B., Stein J. D. (2014). Reduced fluorescein angiography and fundus photography use in the management of neovascular macular degeneration and macular edema during the past decade. *Investigative Ophthalmology & Visual Science*.

[8] Boiko E. V., Maltsev D. S. (2016). Retro-mode scanning laser ophthalmoscopy planning for navigated macular laser photocoagulation in macular edema. *Journal of Ophthalmology*.

[9] Schatz H., Madeira D., McDonald H. R., Johnso R. N. (1991). Progressive enlargement of laser scars following grid laser photocoagulation for diffuse diabetic macular edema. *Archives of Ophthalmology*.

[10] Schmidinger G., Maar N., Bolz M., Scholda C., Schmidt-Erfurth U. (2011). Repeated intravitreal bevacizumab (Avastin(®)) treatment of persistent new vessels in proliferative diabetic retinopathy after complete panretinal photocoagulation. *Acta Ophthalmologica*.

[11] Reznicek L., Cserhati S., Seidensticker F. (2013). Functional and morphological changes in diabetic macular edema over the course of anti-vascular endothelial growth factor treatment. *Acta Ophthalmologica*.

[12] Kozak I., El-Emam S. Y., Cheng L. (2014). Fluorescein angiography versus optical coherence tomography-guided planning for macular laser photocoagulation in diabetic macular edema. *Retina*.

[13] Chylack L. T., Wolfe J. K., Singer D. M. (1993). The lens opacities classification system III. The longitudinal study of cataract study group. *Archives of Ophthalmology*.

[14] Kernt M., Cheuteu R. E., Cserhati S. (2012). Pain and accuracy of focal laser treatment for diabetic macular edema using a retinal navigated laser (Navilas). *Clinical Ophthalmology*.

[15] Kozak I., Oster S. F., Cortes M. A. (2011). Clinical evaluation and treatment accuracy in diabetic macular edema using navigated laser photocoagulator NAVILAS. *Ophthalmology*.

[16] Elman M. J., Aiello L. P., Beck R. W. (2010). Randomized trial evaluating ranibizumab plus prompt or deferred laser or triamcinolone plus prompt laser for diabetic macular edema. *Ophthalmology*.

[17] Diabetic Retinopathy Clinical Research Network, Elman M. J., Qin H. (2012). Intravitreal ranibizumab for diabetic macular edema with prompt versus deferred laser treatment: three-year randomized trial results. *Ophthalmology*.

[18] Brown D. M., Schmidt-Erfurth U., Do D. V. (2015). Intravitreal aflibercept for diabetic macular edema: 100-week results from the VISTA and VIVID studies. *Ophthalmology*.

[19] Chhablani J. K. (2011). Anti-VEGF (vascular endothelial growth factor) drugs in diabetic macular oedema. *Eye*.

